# Penta­kis(l-prolinium) dodeca­tungsto­borate trihydrate

**DOI:** 10.1107/S1600536809047357

**Published:** 2009-11-14

**Authors:** Akbar Raissi Shabari, Mehrdad Pourayoubi, Dena Ghamari

**Affiliations:** a Faculty of Chemistry, Islamic Azad University-North Tehran Branch, Tehran, Iran; b Department of Chemistry, Ferdowsi University of Mashhad, Mashhad, 91779, Iran

## Abstract

The title polyoxometalate-based organic-inorganic hybrid compound, (C_5_H_10_NO_2_)_5_[BW_12_O_40_]·3H_2_O, consists of one α-Keggin-type [BW_12_O_40_]^5−^ polyoxoanion, five crystallographically independent l-prolinium cations and three uncoordin­ated water mol­ecules. The polyoxoanion shows characteristic features with respect to bond lengths and angles. Several N—H⋯O and O—H⋯O hydrogen bonds between the organic cations, inorganic anions and crystal water mol­ecules lead to a three-dimensional supra­molecular structure.

## Related literature

For other [P*X*_12_O_40_]^3−^ (*X* = Mo, W) polyoxometalate anions with organic counter-cations, see: Pourayoubi & Mahjoub (2007 ▶); Pourayoubi *et al.* (2008 ▶). For synthetic details, see: Rocchiccioli-Deltcheff *et al.* (1983 ▶).
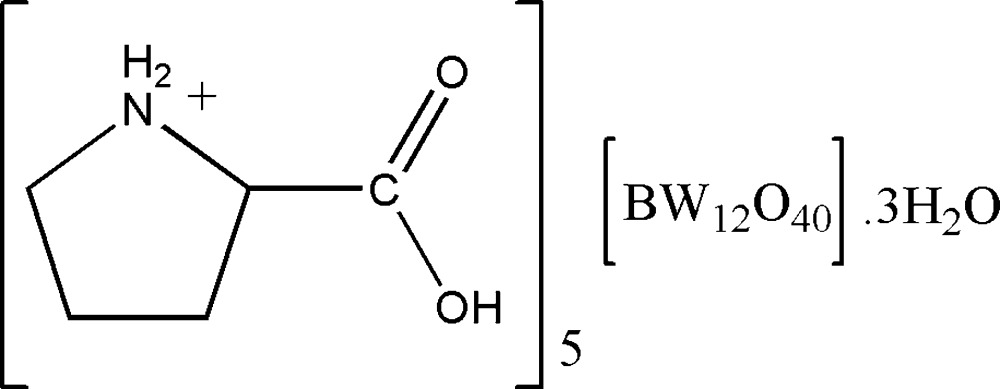


## Experimental

### 

#### Crystal data

(C_5_H_10_NO_2_)_5_[BW_12_O_40_]·3H_2_O
*M* *_r_* = 3491.76
Orthorhombic, 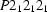

*a* = 12.9135 (3) Å
*b* = 21.6013 (5) Å
*c* = 22.2343 (5) Å
*V* = 6202.2 (2) Å^3^
*Z* = 4
Mo *K*α radiation
μ = 22.27 mm^−1^
*T* = 100 K
0.20 × 0.10 × 0.10 mm

#### Data collection

Bruker SMART APEXII CCD area-detector diffractometer
Absorption correction: multi-scan (*APEX2*; Bruker, 2005 ▶) *T*_min_ = 0.084, *T*_max_ = 0.110
95451 measured reflections
16473 independent reflections
15774 reflections with *I* > 2σ(*I*)
*R*_int_ = 0.078

#### Refinement

*R*[*F* ^2^ > 2σ(*F* ^2^)] = 0.035
*wR*(*F* ^2^) = 0.088
*S* = 1.07
16473 reflections
865 parameters
H-atom parameters constrained
Δρ_max_ = 3.37 e Å^−3^
Δρ_min_ = −1.04 e Å^−3^
Absolute structure: Flack (1983 ▶), 7492 Friedel pairs
Flack parameter: 0.008 (10)

### 

Data collection: *APEX2* (Bruker, 2005 ▶); cell refinement: *SAINT* (Bruker, 2005 ▶); data reduction: *SAINT*; program(s) used to solve structure: *SHELXTL* (Sheldrick, 2008 ▶); program(s) used to refine structure: *SHELXTL*; molecular graphics: *SHELXTL*; software used to prepare material for publication: *SHELXTL*.

## Supplementary Material

Crystal structure: contains datablocks I, global. DOI: 10.1107/S1600536809047357/wm2266sup1.cif

Structure factors: contains datablocks I. DOI: 10.1107/S1600536809047357/wm2266Isup2.hkl

Additional supplementary materials:  crystallographic information; 3D view; checkCIF report

## Figures and Tables

**Table 1:** Hydrogen-bond geometry (Å, °)

*D*—H⋯*A*	*D*—H	H⋯*A*	*D*⋯*A*	*D*—H⋯*A*
N1—H1*NB*⋯O12^i^	0.92	2.47	3.228 (12)	140
N1—H1*NB*⋯O16^i^	0.92	2.51	3.059 (11)	119
N1—H1*NB*⋯O18^i^	0.92	2.49	3.132 (12)	128
N1—H1*NB*⋯O22^i^	0.92	2.28	3.016 (11)	136
O1*S*—H1*O*⋯O38^ii^	0.72	2.18	2.669 (11)	126
N1—H1*NA*⋯O2*S*	0.92	2.24	2.725 (12)	112
N1—H1*NA*⋯O36^iii^	0.92	2.01	2.855 (12)	152
N2—H2*NB*⋯O3*S*	0.92	2.18	2.687 (12)	114
N2—H2*NB*⋯O40^iv^	0.92	1.93	2.744 (11)	146
N2—H2*NA*⋯O6^v^	0.92	1.90	2.810 (11)	170
O4*S*—H4*O*⋯O3*W*	0.92	1.73	2.647 (12)	170
N3—H3*NB*⋯O3*S* ^ii^	0.92	2.18	2.909 (13)	135
N3—H3*NA*⋯O5*S*	0.92	2.16	2.672 (11)	114
N3—H3*NA*⋯O10*S*	0.92	2.05	2.856 (12)	145
O6*S*—H6*O*⋯O1*W*	0.84	1.88	2.638 (13)	149
N4—H4*NB*⋯O7	0.92	1.96	2.821 (13)	155
O7*S*—H7*O*⋯O21^vi^	0.84	1.87	2.689 (11)	165
N4—H4*NA*⋯O8*S*	0.92	2.29	2.701 (13)	107
N5—H5*NB*⋯O10*S*	0.92	2.26	2.709 (12)	109
N5—H5*NB*⋯O25^i^	0.92	2.20	3.005 (11)	146
O9*S*—H9*O*⋯O2*W*	0.89	1.86	2.651 (12)	147
N5—H5*NA*⋯O31	0.92	2.25	2.830 (12)	120
N5—H5*NA*⋯O8*S* ^vii^	0.92	2.00	2.855 (13)	154

Symmetry codes: (i) 
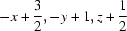
; (ii) 
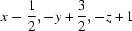
; (iii) 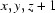; (iv) 
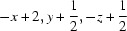
; (v) 
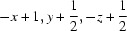
; (vi) 
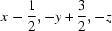
; (vii) 
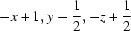
.

**Fig. 1.**
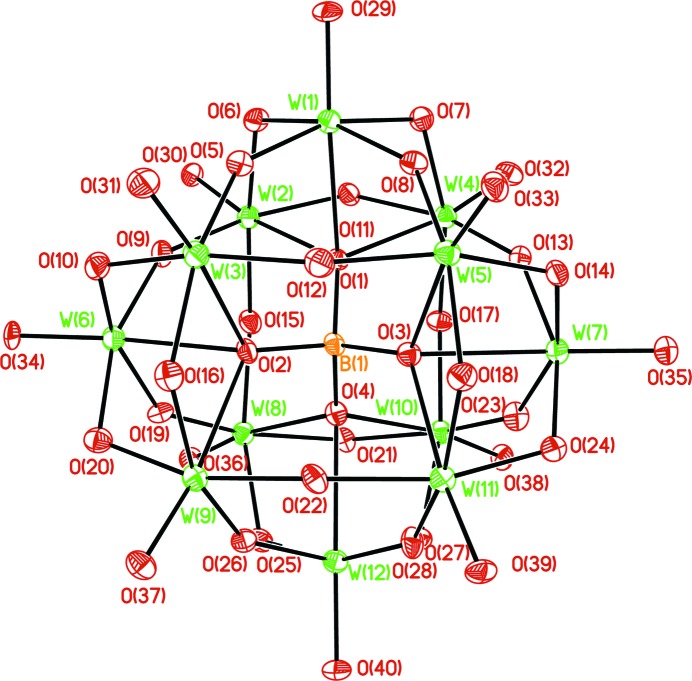
An ORTEP-style plot of the α-Keggin-type BW12O405- polyoxoanion. Ellipsoids are given at the 30% probability level.

**Fig. 2.**
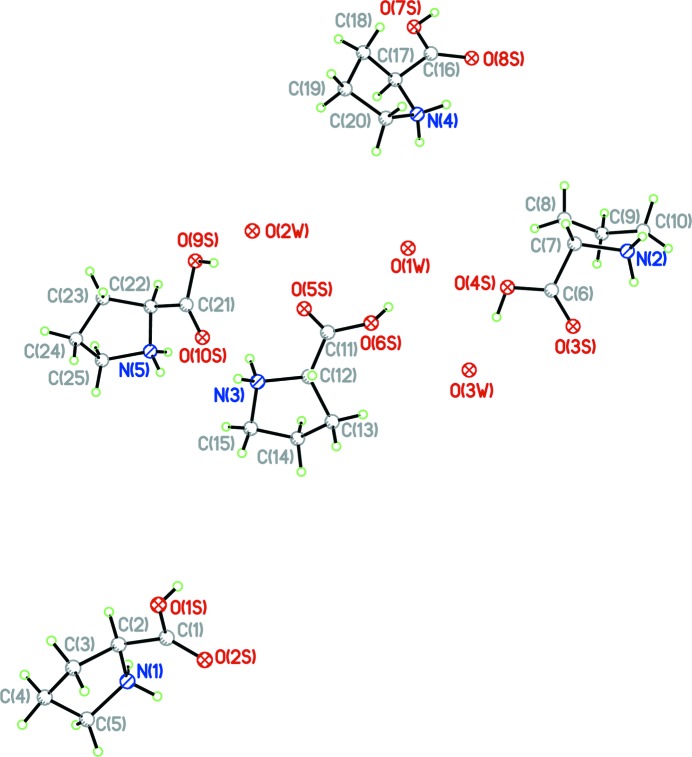
A view of the five symmetrically independent organic cations and the crystal water molecules. Ellipsoids are given at the 30% probability level.

## supplementary crystallographic information

### Comment

Continuing our previous studies on synthesis and X-ray structure determination of some P*X*_12_O_40_^3-^-based (*X* = Mo, W) organic inorganic hybrid compounds such as (C_8_H_10_N_5_)[H_2_PMo_12_O_40_].2.5CH_3_OH.4H_2_O (Pourayoubi & Mahjoub, 2007) and [CH_3_CH_2_C(OH)^+^NHCH_3_]_3_[PW_12_O_40_].0.5CH_3_CH_2_C(O)NHCH_3_.2H_2_O (Pourayoubi *et al.*, 2008), we report here on the synthesis and crystal structure of a new hybrid material containing a [BW_12_O_40_]^5-^ polyoxoanion.

The title polyoxometalate-based organic-inorganic hybrid compound consists of one [BW_12_O_40_]^5-^ polyoxoanion, five crystallographically independent L-prolinium cations and three crystal water molecules. The inorganic anion shows a classical α-Keggin structure (Fig. 1) with 4 different types of O atoms. This includes 12 terminal O atoms, 4 O atoms that are bonded to B and W atoms, 12 corner-sharing and 12 edge-sharing O atoms that both are part of distorted WO_6_ octahedra. The central BO_4_ tetrahedron is slightly distorted and is surrounded by 12 distorted WO_6_ octahedra. The B—O bond lengths range from 1.503 (12) to 1.556 (12) Å, and the O—B—O angles are in the range of 108.0 (7)–111.0 (7)°.

All five organic cations (Fig. 2) show slight differences in bond lengths, angles and torsion angles. They are involved in an extensive hydrogen bonding. Several N—H···O (N···O distances are in the range of 2.672 (11) to 3.228 (12) Å) and O—H···O (O···O distances are in the range from 2.638 (13) to 2.689 (11) Å) hydrogen bonds between the organic cations, inorganic anions and crystal water molecules lead to a 3-D supramolecular framework.

### Experimental

Synthesis of K_5_BW_12_O_40_^.^*n*H_2_O: K_5_BW_12_O_40_^.^*n*H_2_O was prepared according to the literature method by Rocchiccioli-Deltcheff *et al.* (1983). To 100 ml of water were added successively under vigorous stirring 100 g of Na_2_WO_4_^.^2H_2_O, 5 g of boric acid (H_3_BO_3_), and 60 ml of aqueous 6*M* HCl. The solution (pH=6) was allowed to boil for some hours, with water re-filled from time to time. The solid paratungstate Na_l0_W_12_O_41_^.^*n*H_2_O (17 g) was filtered off; the filtrate was then acidified by aqueous 6*M* HCl to pH=2 and allowed to boil for another 0.5 h. Precipitation of K_5_BW_12_O_40_.*n*H_2_O was accomplished by adding 20 g of solid KCl. After filtration and washing with Et_2_O, 71 g of crude product were obtained and recrystallized in 50 ml of water at 333 K. ^11^B-NMR (D_2_O, p.p.m.):-17.45. IR (KBr, cm^-1^): 3420 *s*, 1942 *s*, 1603 *s*, 959 *vs*, 904 *vs*, 811 *vs*, 506 *m*, 376 *m*. UV-Vis (1.2 × 10^-4^*M* in water, λ_max_): 222 nm, 256 nm.

Synthesis of H_5_BW_12_O_40_^.^*y*H_2_O: The acid was prepared by passage of an aqueous solution of K_5_BW_12_O_40_^.^*n*H_2_O through a column (50 cm×1 cm) of DOWEX-50 W-X8 (H^+^ enriched cationic exchange resin) and subsequent evaporation of the solution to dryness. ^11^B-NMR (D_2_O, p.p.m.): -17.54. IR (KBr, cm^-1^): 3095*b*, 1702*m*, 1154 *s*, 796 *vs*, 529 *m*, 383 *m*. UV-Vis (1.2 × 10^-4^*M* in water, λ_max_): 223 nm, 255 nm.

Synthesis of [C_5_H_10_NO_2_]_5_[BW_12_O_40_].3H_2_O: A solution of L-proline (0.302 g, 2.62 mmol) in HCl (15 ml, 1 *M*) was added dropwise to a solution of H_5_BW_12_O_40_.yH_2_O (1.5 g) in H_2_O (60 ml) and stirred for 2 h. The solution was filtered and kept until colorless crystals were obtained. ^11^B-NMR (D_2_O, p.p.m.): -17.45. IR (KBr, cm^-1^): 2928 *m*, 1739 *m*, 1579 *m*, 1456 *m*, 1369 *m*, 1240 *m*, 960 *s*, 907 *s*, 807 *vs*, 499 *m*, 425 *w*, 390 *w*. ^1^H-NMR (D_2_O, p.p.m.): 2.17 (*m*), 2.52 (*m*), 3.50 (*m*), 4.47 (*m*). ^13^C-NMR (D_2_O, p.p.m.): 23.14, 28.01, 46.21, 59.43, 171.52. UV-Vis (1.2 × 10^-4^*M* in water, λ_max_): 222 nm, 256 nm.

### Refinement

The hydrogen atoms of organic OH and NH_2_ groups were found in difference Fourier synthesis and were fixed. Water H atoms could not be located unambiguously and were excluded from the refinement. The H(C) atom positions of the organic cation were calculated. All hydrogen atoms were refined in an isotropic approximation using a riding model with *U*_iso_(H) equal to 1.2 *U*_eq_(Ci), for methyl groups equal to 1.5 *U*_eq_(Cii), where U(Ci) and U(Cii) are respectively the equivalent thermal parameters of the carbon atoms to which the corresponding H atoms are bonded. The highest peak and the deepest hole in the final Fourier map are located 0.75 Å from atom W1 and 1.38 from atom O19, respectively.

### Crystal data

**Table:**

(C_5_H_10_NO_2_)_5_[BW_12_O_40_]·3H_2_O	*F*(000) = 6232
*M**_r_* = 3491.76	*D*_x_ = 3.739 Mg m^−^^3^
Orthorhombic, *P*2_1_2_1_2_1_	Mo *K*α radiation, λ = 0.71073 Å
Hall symbol: P 2ac 2ab	Cell parameters from 1485 reflections
*a* = 12.9135 (3) Å	θ = 2–25°
*b* = 21.6013 (5) Å	µ = 22.27 mm^−^^1^
*c* = 22.2343 (5) Å	*T* = 100 K
*V* = 6202.2 (2) Å^3^	Prism, colorless
*Z* = 4	0.20 × 0.10 × 0.10 mm

### Data collection

**Table:**

Bruker SMART APEXII CCD area-detector diffractometer	16473 independent reflections
Radiation source: fine-focus sealed tube	15774 reflections with *I* > 2σ(*I*)
graphite	*R*_int_ = 0.078
φ and ω scans	θ_max_ = 29.0°, θ_min_ = 1.3°
Absorption correction: multi-scan (*APEX2*; Bruker, 2005)	*h* = −17→17
*T*_min_ = 0.084, *T*_max_ = 0.110	*k* = −29→29
95451 measured reflections	*l* = −30→30

### Refinement

**Table:**

Refinement on *F*^2^	Secondary atom site location: difference Fourier map
Least-squares matrix: full	Hydrogen site location: inferred from neighbouring sites
*R*[*F*^2^ > 2σ(*F*^2^)] = 0.035	H-atom parameters constrained
*wR*(*F*^2^) = 0.088	*w* = 1/[σ^2^(*F*_o_^2^) + (0.06*P*)^2^ + 1.7105*P*] where *P* = (*F*_o_^2^ + 2*F*_c_^2^)/3
*S* = 1.07	(Δ/σ)_max_ = 0.025
16473 reflections	Δρ_max_ = 3.37 e Å^−^^3^
865 parameters	Δρ_min_ = −1.04 e Å^−^^3^
0 restraints	Absolute structure: Flack (1983), 7492 Friedel pairs
Primary atom site location: structure-invariant direct methods	Flack parameter: 0.008 (10)

### Special details

**Table:**

Geometry. All e.s.d.'s (except the e.s.d. in the dihedral angle between two l.s. planes) are estimated using the full covariance matrix. The cell e.s.d.'s are taken into account individually in the estimation of e.s.d.'s in distances, angles and torsion angles; correlations between e.s.d.'s in cell parameters are only used when they are defined by crystal symmetry. An approximate (isotropic) treatment of cell e.s.d.'s is used for estimating e.s.d.'s involving l.s. planes.
Refinement. Refinement of *F*^2^ against ALL reflections. The weighted *R*-factor *wR* and goodness of fit *S* are based on *F*^2^, conventional *R*-factors *R* are based on *F*, with *F* set to zero for negative *F*^2^. The threshold expression of *F*^2^ > σ(*F*^2^) is used only for calculating *R*-factors(gt) *etc*. and is not relevant to the choice of reflections for refinement. *R*-factors based on *F*^2^ are statistically about twice as large as those based on *F*, and *R*- factors based on ALL data will be even larger.

### Fractional atomic coordinates and isotropic or equivalent isotropic displacement parameters (Å^2^)

**Table:**

	*x*	*y*	*z*	*U*_iso_*/*U*_eq_	
W1	0.48790 (3)	0.656796 (18)	0.276922 (17)	0.02402 (8)	
W2	0.51869 (3)	0.622545 (18)	0.131253 (17)	0.02457 (8)	
W3	0.63191 (3)	0.520433 (18)	0.326329 (17)	0.02592 (8)	
W4	0.59752 (3)	0.757894 (18)	0.183500 (17)	0.02368 (8)	
W5	0.71404 (3)	0.674877 (18)	0.373609 (16)	0.02460 (8)	
W6	0.65206 (3)	0.482129 (17)	0.183055 (17)	0.02480 (8)	
W7	0.82724 (3)	0.771149 (18)	0.277591 (17)	0.02396 (8)	
W8	0.77701 (3)	0.584912 (18)	0.075721 (17)	0.02402 (8)	
W9	0.86311 (3)	0.491233 (18)	0.266892 (17)	0.02491 (8)	
W10	0.85105 (3)	0.725253 (18)	0.120431 (16)	0.02382 (8)	
W11	0.94839 (3)	0.643680 (18)	0.320518 (17)	0.02371 (8)	
W12	0.98281 (3)	0.601157 (18)	0.163090 (16)	0.02424 (8)	
B1	0.7377 (8)	0.6289 (5)	0.2248 (4)	0.0200 (17)	
O1	0.6302 (5)	0.6553 (3)	0.2105 (3)	0.0210 (12)	
O1S	0.4293 (6)	0.5931 (4)	0.9191 (4)	0.0352 (17)	
H1O	0.4195	0.6182	0.8988	0.042*	
O1W	0.4607 (8)	0.7964 (4)	0.3734 (4)	0.0404 (19)	
O2	0.7256 (6)	0.5594 (3)	0.2420 (3)	0.0222 (12)	
O2W	0.2460 (6)	0.6391 (4)	0.4227 (4)	0.0350 (16)	
O2S	0.6010 (7)	0.6037 (4)	0.9327 (4)	0.0382 (18)	
O3	0.7868 (5)	0.6627 (3)	0.2765 (3)	0.0231 (12)	
O3W	0.7275 (7)	0.8309 (4)	0.4577 (4)	0.0359 (16)	
O3S	0.7876 (6)	0.9536 (4)	0.3750 (4)	0.0349 (16)	
O4	0.8061 (5)	0.6326 (3)	0.1696 (3)	0.0241 (13)	
O4S	0.6491 (6)	0.8905 (4)	0.3642 (4)	0.0338 (16)	
H4O	0.6769	0.8738	0.3989	0.041*	
O5	0.5257 (6)	0.5769 (3)	0.2986 (3)	0.0271 (14)	
O5S	0.4451 (6)	0.6644 (4)	0.4605 (3)	0.0342 (16)	
O6	0.4292 (5)	0.6287 (3)	0.2023 (3)	0.0251 (13)	
O6S	0.4772 (8)	0.7641 (4)	0.4874 (4)	0.0391 (18)	
H6O	0.4961	0.7757	0.4531	0.047*	
O7	0.4925 (6)	0.7394 (3)	0.2423 (3)	0.0256 (13)	
O7S	0.2860 (8)	0.7803 (4)	0.0535 (4)	0.0416 (19)	
H7O	0.2990	0.8017	0.0229	0.062*	
O8	0.5863 (5)	0.6868 (3)	0.3341 (3)	0.0236 (13)	
O8S	0.3474 (7)	0.8638 (4)	0.1020 (4)	0.0356 (17)	
O9	0.5532 (6)	0.5404 (3)	0.1617 (3)	0.0267 (14)	
O9S	0.3117 (7)	0.5230 (4)	0.4169 (4)	0.0416 (19)	
H9O	0.3033	0.5608	0.4324	0.050*	
O10	0.5915 (6)	0.4682 (3)	0.2596 (3)	0.0299 (15)	
O10S	0.4223 (7)	0.5205 (4)	0.4950 (4)	0.0389 (18)	
O11	0.5160 (5)	0.7088 (3)	0.1264 (3)	0.0250 (13)	
O12	0.6896 (6)	0.5883 (4)	0.3686 (3)	0.0314 (15)	
O13	0.6928 (5)	0.7809 (3)	0.2427 (3)	0.0241 (13)	
O14	0.7592 (6)	0.7557 (3)	0.3529 (3)	0.0241 (13)	
O15	0.6373 (6)	0.6135 (3)	0.0844 (3)	0.0268 (14)	
O16	0.7606 (6)	0.4786 (3)	0.3293 (3)	0.0292 (14)	
O17	0.7111 (6)	0.7484 (3)	0.1301 (3)	0.0269 (14)	
O18	0.8576 (6)	0.6533 (4)	0.3872 (3)	0.0284 (14)	
O19	0.7369 (5)	0.5154 (3)	0.1199 (3)	0.0251 (13)	
O20	0.7781 (6)	0.4446 (4)	0.2136 (3)	0.0279 (14)	
O21	0.8143 (6)	0.6694 (3)	0.0559 (3)	0.0248 (13)	
O22	0.9096 (6)	0.5585 (3)	0.3152 (3)	0.0271 (14)	
O23	0.8733 (6)	0.7575 (4)	0.1983 (3)	0.0302 (15)	
O24	0.9457 (5)	0.7318 (3)	0.3100 (3)	0.0240 (13)	
O25	0.9204 (6)	0.5700 (3)	0.0899 (3)	0.0273 (14)	
O26	0.9358 (6)	0.5286 (3)	0.2028 (3)	0.0268 (14)	
O27	0.9791 (5)	0.6809 (3)	0.1284 (3)	0.0246 (13)	
O28	0.9970 (6)	0.6367 (3)	0.2391 (3)	0.0292 (15)	
O29	0.3749 (5)	0.6661 (4)	0.3156 (3)	0.0285 (14)	
O30	0.4232 (5)	0.6030 (4)	0.0816 (3)	0.0285 (15)	
O31	0.5646 (6)	0.4846 (4)	0.3829 (4)	0.0347 (16)	
O32	0.5517 (6)	0.8289 (4)	0.1618 (3)	0.0297 (15)	
O33	0.6819 (6)	0.6906 (3)	0.4468 (3)	0.0278 (14)	
O34	0.6062 (6)	0.4176 (3)	0.1473 (3)	0.0293 (15)	
O35	0.8625 (6)	0.8463 (3)	0.2907 (3)	0.0290 (14)	
O36	0.7750 (6)	0.5562 (4)	0.0047 (3)	0.0280 (14)	
O37	0.9478 (6)	0.4343 (3)	0.2885 (3)	0.0286 (14)	
O38	0.8953 (6)	0.7851 (3)	0.0763 (3)	0.0275 (14)	
O39	1.0646 (5)	0.6363 (4)	0.3586 (3)	0.0272 (14)	
O40	1.1101 (5)	0.5826 (4)	0.1480 (3)	0.0284 (14)	
N1	0.6289 (7)	0.4792 (4)	0.9439 (4)	0.0297 (17)	
H1NB	0.6520	0.4592	0.9099	0.036*	
H1NA	0.6732	0.5116	0.9517	0.036*	
N2	0.7218 (7)	1.0352 (4)	0.2922 (4)	0.0272 (16)	
H2NB	0.7719	1.0375	0.3215	0.033*	
H2NA	0.6776	1.0682	0.2966	0.033*	
N3	0.4500 (8)	0.6235 (4)	0.5739 (4)	0.0320 (18)	
H3NB	0.3905	0.6209	0.5965	0.038*	
H3NA	0.4383	0.6041	0.5377	0.038*	
N4	0.3201 (8)	0.8133 (5)	0.2120 (4)	0.035 (2)	
H4NB	0.3851	0.8006	0.2236	0.041*	
H4NA	0.3224	0.8549	0.2034	0.041*	
N5	0.4818 (7)	0.4030 (4)	0.4701 (4)	0.0284 (17)	
H5NB	0.4970	0.4261	0.5036	0.034*	
H5NA	0.5381	0.4035	0.4447	0.034*	
C1	0.5225 (10)	0.5737 (5)	0.9283 (5)	0.033 (2)	
C2	0.5198 (8)	0.5038 (5)	0.9335 (4)	0.0283 (19)	
H2A	0.4914	0.4858	0.8954	0.034*	
C3	0.4581 (9)	0.4787 (5)	0.9872 (5)	0.033 (2)	
H3A	0.3853	0.4703	0.9757	0.040*	
H3B	0.4589	0.5084	1.0211	0.040*	
C4	0.5143 (9)	0.4184 (5)	1.0041 (5)	0.030 (2)	
H4A	0.4996	0.4065	1.0462	0.036*	
H4B	0.4941	0.3841	0.9771	0.036*	
C5	0.6279 (9)	0.4355 (5)	0.9958 (5)	0.032 (2)	
H5A	0.6556	0.4556	1.0325	0.038*	
H5B	0.6699	0.3983	0.9870	0.038*	
C6	0.7076 (9)	0.9376 (5)	0.3491 (5)	0.031 (2)	
C7	0.6617 (8)	0.9738 (5)	0.2975 (5)	0.0289 (19)	
H7A	0.5862	0.9815	0.3039	0.035*	
C8	0.6816 (10)	0.9420 (5)	0.2356 (5)	0.035 (2)	
H8A	0.6867	0.8966	0.2403	0.042*	
H8B	0.6251	0.9515	0.2070	0.042*	
C9	0.7846 (9)	0.9690 (5)	0.2134 (5)	0.031 (2)	
H9A	0.8443	0.9503	0.2346	0.037*	
H9B	0.7931	0.9632	0.1695	0.037*	
C10	0.7721 (9)	1.0363 (5)	0.2293 (5)	0.031 (2)	
H10A	0.7269	1.0577	0.1999	0.037*	
H10B	0.8401	1.0575	0.2305	0.037*	
C11	0.4650 (8)	0.7049 (5)	0.4984 (5)	0.030 (2)	
C12	0.4773 (9)	0.6902 (5)	0.5634 (4)	0.0281 (19)	
H12A	0.4310	0.7175	0.5879	0.034*	
C13	0.5897 (9)	0.6957 (5)	0.5863 (4)	0.031 (2)	
H13A	0.5915	0.7129	0.6275	0.037*	
H13B	0.6312	0.7227	0.5595	0.037*	
C14	0.6314 (10)	0.6283 (6)	0.5852 (5)	0.037 (2)	
H14A	0.6529	0.6158	0.5443	0.045*	
H14B	0.6902	0.6229	0.6133	0.045*	
C15	0.5367 (9)	0.5927 (6)	0.6060 (5)	0.036 (2)	
H15A	0.5417	0.5485	0.5946	0.043*	
H15B	0.5279	0.5958	0.6501	0.043*	
C16	0.3092 (9)	0.8113 (5)	0.1013 (5)	0.035 (2)	
C17	0.2839 (9)	0.7769 (6)	0.1577 (5)	0.034 (2)	
H17A	0.3215	0.7364	0.1569	0.041*	
C18	0.1722 (11)	0.7646 (9)	0.1700 (7)	0.057 (4)	
H18A	0.1477	0.7275	0.1481	0.068*	
H18B	0.1288	0.8005	0.1587	0.068*	
C19	0.1709 (14)	0.7542 (11)	0.2365 (7)	0.071 (6)	
H19A	0.1002	0.7602	0.2528	0.085*	
H19B	0.1945	0.7118	0.2463	0.085*	
C20	0.2427 (10)	0.8008 (5)	0.2611 (6)	0.037 (2)	
H20A	0.2777	0.7847	0.2975	0.044*	
H20B	0.2050	0.8392	0.2719	0.044*	
C21	0.3792 (9)	0.4961 (5)	0.4548 (5)	0.032 (2)	
C22	0.3904 (11)	0.4276 (5)	0.4397 (7)	0.044 (3)	
H22A	0.3974	0.4221	0.3952	0.053*	
C23	0.2989 (12)	0.3902 (8)	0.4629 (12)	0.075 (6)	
H23A	0.2485	0.4177	0.4832	0.090*	
H23B	0.2635	0.3690	0.4292	0.090*	
C24	0.3413 (10)	0.3438 (7)	0.5063 (6)	0.045 (3)	
H24A	0.3041	0.3039	0.5029	0.054*	
H24B	0.3356	0.3591	0.5482	0.054*	
C25	0.4547 (9)	0.3368 (5)	0.4877 (5)	0.034 (2)	
H25A	0.4980	0.3222	0.5216	0.041*	
H25B	0.4623	0.3081	0.4533	0.041*	

### Atomic displacement parameters (Å^2^)

**Table:**

	*U*^11^	*U*^22^	*U*^33^	*U*^12^	*U*^13^	*U*^23^
W1	0.02403 (17)	0.02409 (17)	0.02394 (16)	−0.00038 (13)	0.00101 (14)	−0.00086 (13)
W2	0.02475 (17)	0.02495 (17)	0.02402 (16)	−0.00076 (14)	−0.00093 (14)	−0.00166 (13)
W3	0.02953 (19)	0.02317 (17)	0.02507 (16)	0.00055 (14)	0.00420 (14)	0.00201 (13)
W4	0.02451 (17)	0.02244 (17)	0.02408 (16)	0.00017 (13)	−0.00103 (14)	0.00037 (13)
W5	0.02514 (17)	0.02672 (18)	0.02194 (15)	0.00076 (14)	0.00060 (14)	−0.00149 (13)
W6	0.02673 (18)	0.02187 (17)	0.02580 (16)	−0.00172 (14)	0.00042 (14)	−0.00100 (13)
W7	0.02653 (18)	0.02181 (16)	0.02353 (15)	−0.00008 (13)	−0.00197 (14)	−0.00132 (13)
W8	0.02555 (18)	0.02436 (17)	0.02216 (15)	−0.00149 (14)	0.00052 (14)	−0.00160 (13)
W9	0.02780 (18)	0.02266 (16)	0.02428 (16)	0.00153 (14)	0.00033 (14)	0.00044 (13)
W10	0.02612 (17)	0.02229 (16)	0.02304 (15)	−0.00099 (14)	0.00081 (14)	0.00083 (13)
W11	0.02437 (17)	0.02433 (17)	0.02243 (15)	0.00041 (13)	−0.00105 (13)	0.00032 (13)
W12	0.02416 (17)	0.02574 (18)	0.02283 (15)	0.00163 (14)	0.00070 (14)	0.00029 (13)
B1	0.026 (5)	0.018 (4)	0.016 (4)	−0.001 (3)	0.006 (3)	−0.001 (3)
O1	0.023 (3)	0.019 (3)	0.021 (3)	0.000 (2)	0.007 (2)	−0.002 (2)
O1S	0.036 (4)	0.030 (4)	0.039 (4)	0.004 (3)	−0.003 (3)	0.006 (3)
O1W	0.056 (5)	0.029 (4)	0.037 (4)	0.004 (4)	0.005 (4)	0.004 (3)
O2	0.029 (3)	0.015 (3)	0.023 (3)	0.001 (2)	0.005 (3)	0.000 (2)
O2W	0.028 (4)	0.044 (4)	0.033 (4)	−0.001 (3)	−0.003 (3)	−0.004 (3)
O2S	0.036 (4)	0.034 (4)	0.045 (4)	−0.004 (3)	−0.003 (4)	0.001 (3)
O3	0.024 (3)	0.025 (3)	0.020 (3)	0.001 (3)	0.000 (2)	−0.002 (2)
O3W	0.040 (4)	0.037 (4)	0.031 (3)	−0.001 (3)	0.002 (3)	−0.002 (3)
O3S	0.032 (4)	0.030 (4)	0.043 (4)	−0.003 (3)	−0.001 (3)	−0.002 (3)
O4	0.027 (3)	0.022 (3)	0.023 (3)	0.000 (2)	0.000 (3)	−0.002 (2)
O4S	0.033 (4)	0.032 (4)	0.037 (4)	0.001 (3)	0.006 (3)	0.004 (3)
O5	0.026 (3)	0.027 (3)	0.028 (3)	0.001 (3)	0.001 (3)	−0.001 (3)
O5S	0.034 (4)	0.041 (4)	0.027 (3)	−0.007 (3)	0.001 (3)	−0.001 (3)
O6	0.021 (3)	0.032 (4)	0.023 (3)	0.000 (3)	−0.006 (2)	−0.003 (3)
O6S	0.056 (5)	0.030 (4)	0.032 (4)	0.002 (4)	−0.001 (4)	0.006 (3)
O7	0.026 (3)	0.023 (3)	0.028 (3)	0.001 (3)	0.000 (3)	0.003 (2)
O7S	0.055 (5)	0.040 (4)	0.030 (4)	−0.010 (4)	−0.003 (4)	0.005 (3)
O8	0.022 (3)	0.028 (3)	0.021 (3)	0.004 (2)	−0.002 (2)	0.003 (2)
O8S	0.036 (4)	0.033 (4)	0.038 (4)	−0.007 (3)	0.000 (3)	0.002 (3)
O9	0.030 (3)	0.021 (3)	0.028 (3)	−0.002 (3)	−0.003 (3)	−0.004 (3)
O9S	0.047 (5)	0.032 (4)	0.046 (4)	0.006 (4)	−0.008 (4)	0.000 (4)
O10	0.033 (4)	0.026 (3)	0.031 (3)	−0.006 (3)	0.010 (3)	0.000 (3)
O10S	0.051 (5)	0.032 (4)	0.034 (4)	0.004 (4)	−0.008 (4)	−0.005 (3)
O11	0.026 (3)	0.022 (3)	0.027 (3)	0.003 (3)	−0.002 (3)	−0.003 (2)
O12	0.031 (4)	0.031 (4)	0.032 (3)	0.002 (3)	0.002 (3)	0.000 (3)
O13	0.022 (3)	0.022 (3)	0.028 (3)	−0.001 (2)	−0.001 (3)	−0.003 (2)
O14	0.028 (3)	0.019 (3)	0.024 (3)	0.000 (3)	0.002 (3)	−0.004 (2)
O15	0.032 (4)	0.024 (3)	0.024 (3)	0.001 (3)	0.004 (3)	−0.004 (2)
O16	0.033 (4)	0.028 (3)	0.027 (3)	0.001 (3)	−0.002 (3)	0.003 (3)
O17	0.025 (3)	0.032 (4)	0.023 (3)	0.002 (3)	0.008 (3)	0.000 (3)
O18	0.027 (3)	0.031 (4)	0.027 (3)	0.002 (3)	−0.005 (3)	0.001 (3)
O19	0.017 (3)	0.032 (3)	0.026 (3)	−0.001 (3)	0.002 (2)	0.002 (3)
O20	0.027 (3)	0.032 (4)	0.025 (3)	0.001 (3)	0.004 (3)	0.001 (3)
O21	0.031 (4)	0.020 (3)	0.023 (3)	0.002 (3)	−0.002 (3)	−0.002 (2)
O22	0.034 (4)	0.020 (3)	0.027 (3)	0.006 (3)	−0.002 (3)	0.002 (3)
O23	0.038 (4)	0.030 (4)	0.023 (3)	−0.002 (3)	0.000 (3)	0.001 (3)
O24	0.025 (3)	0.028 (3)	0.020 (3)	0.000 (3)	−0.001 (2)	−0.001 (2)
O25	0.028 (3)	0.029 (4)	0.025 (3)	0.007 (3)	−0.004 (3)	−0.004 (3)
O26	0.024 (3)	0.027 (3)	0.030 (3)	−0.002 (3)	0.009 (3)	−0.001 (3)
O27	0.022 (3)	0.025 (3)	0.026 (3)	0.002 (3)	−0.001 (3)	0.001 (2)
O28	0.041 (4)	0.025 (3)	0.022 (3)	−0.006 (3)	0.003 (3)	−0.007 (2)
O29	0.023 (3)	0.036 (4)	0.027 (3)	0.000 (3)	0.011 (3)	0.001 (3)
O30	0.021 (3)	0.031 (4)	0.033 (3)	−0.003 (3)	−0.009 (3)	−0.013 (3)
O31	0.037 (4)	0.034 (4)	0.034 (4)	−0.001 (3)	0.012 (3)	0.005 (3)
O32	0.030 (4)	0.034 (4)	0.025 (3)	0.009 (3)	−0.001 (3)	0.000 (3)
O33	0.033 (4)	0.026 (3)	0.024 (3)	−0.001 (3)	0.003 (3)	−0.005 (3)
O34	0.033 (4)	0.018 (3)	0.037 (4)	−0.009 (3)	0.000 (3)	−0.011 (3)
O35	0.034 (4)	0.023 (3)	0.030 (3)	−0.002 (3)	0.000 (3)	−0.001 (3)
O36	0.030 (4)	0.031 (4)	0.023 (3)	−0.001 (3)	0.001 (3)	−0.004 (3)
O37	0.032 (4)	0.027 (3)	0.026 (3)	0.008 (3)	−0.005 (3)	0.003 (3)
O38	0.035 (4)	0.018 (3)	0.029 (3)	−0.004 (3)	0.006 (3)	0.010 (3)
O39	0.018 (3)	0.037 (4)	0.026 (3)	0.004 (3)	−0.006 (3)	0.000 (3)
O40	0.019 (3)	0.035 (4)	0.031 (3)	0.004 (3)	0.005 (3)	0.001 (3)
N1	0.031 (4)	0.032 (4)	0.027 (4)	0.000 (3)	−0.001 (3)	−0.006 (3)
N2	0.024 (4)	0.026 (4)	0.031 (4)	0.000 (3)	−0.001 (3)	0.003 (3)
N3	0.033 (5)	0.032 (5)	0.030 (4)	0.002 (4)	0.004 (4)	−0.002 (3)
N4	0.032 (5)	0.043 (5)	0.028 (4)	−0.005 (4)	−0.004 (4)	0.001 (4)
N5	0.027 (4)	0.036 (5)	0.022 (3)	0.000 (4)	−0.001 (3)	0.001 (3)
C1	0.042 (6)	0.031 (5)	0.025 (4)	0.006 (4)	−0.001 (4)	−0.002 (4)
C2	0.029 (5)	0.029 (5)	0.027 (4)	0.000 (4)	0.000 (4)	0.000 (4)
C3	0.041 (6)	0.022 (5)	0.038 (5)	0.005 (4)	0.012 (5)	−0.002 (4)
C4	0.037 (5)	0.022 (4)	0.032 (5)	0.006 (4)	0.000 (4)	0.004 (4)
C5	0.036 (5)	0.032 (5)	0.027 (4)	−0.002 (4)	−0.004 (4)	0.000 (4)
C6	0.030 (5)	0.031 (5)	0.032 (5)	0.001 (4)	0.008 (4)	−0.006 (4)
C7	0.028 (5)	0.023 (4)	0.036 (5)	−0.004 (4)	−0.004 (4)	−0.006 (4)
C8	0.050 (7)	0.024 (5)	0.032 (5)	0.005 (4)	−0.006 (5)	−0.012 (4)
C9	0.033 (5)	0.031 (5)	0.030 (4)	0.006 (4)	−0.003 (4)	0.002 (4)
C10	0.033 (5)	0.030 (5)	0.029 (4)	−0.002 (4)	0.003 (4)	0.000 (4)
C11	0.020 (4)	0.041 (6)	0.029 (4)	−0.001 (4)	−0.001 (4)	0.002 (4)
C12	0.036 (5)	0.024 (4)	0.025 (4)	0.004 (4)	0.004 (4)	−0.004 (3)
C13	0.032 (5)	0.041 (6)	0.021 (4)	−0.001 (4)	−0.001 (4)	−0.001 (4)
C14	0.038 (6)	0.044 (6)	0.029 (5)	0.007 (5)	0.006 (4)	0.000 (4)
C15	0.041 (6)	0.035 (6)	0.030 (5)	0.009 (5)	0.004 (4)	0.002 (4)
C16	0.035 (5)	0.029 (5)	0.041 (5)	0.005 (4)	0.000 (5)	0.005 (4)
C17	0.030 (5)	0.045 (6)	0.028 (4)	−0.004 (5)	0.000 (4)	0.002 (4)
C18	0.038 (7)	0.088 (12)	0.044 (7)	−0.020 (7)	−0.005 (6)	−0.001 (7)
C19	0.055 (9)	0.109 (15)	0.047 (8)	−0.042 (10)	0.005 (7)	0.003 (9)
C20	0.042 (6)	0.027 (5)	0.042 (6)	−0.002 (4)	0.017 (5)	0.003 (4)
C21	0.033 (5)	0.031 (5)	0.031 (5)	−0.001 (4)	0.004 (4)	0.000 (4)
C22	0.052 (7)	0.024 (5)	0.057 (7)	−0.003 (5)	−0.021 (6)	0.006 (5)
C23	0.035 (7)	0.044 (8)	0.148 (19)	−0.004 (6)	−0.005 (10)	0.016 (10)
C24	0.035 (6)	0.060 (8)	0.041 (6)	−0.012 (6)	−0.004 (5)	0.010 (6)
C25	0.041 (6)	0.029 (5)	0.031 (5)	−0.004 (4)	−0.004 (4)	0.004 (4)

### Geometric parameters (Å, °)

**Table:**

W1—O29	1.706 (7)	O6S—C11	1.312 (14)
W1—O5	1.857 (7)	O6S—H6O	0.8390
W1—O8	1.911 (7)	O7S—C16	1.292 (15)
W1—O6	1.924 (6)	O7S—H7O	0.8400
W1—O7	1.945 (7)	O8S—C16	1.235 (14)
W1—O1	2.357 (6)	O9S—C21	1.344 (14)
W2—O30	1.708 (6)	O9S—H9O	0.8951
W2—O15	1.863 (7)	O10S—C21	1.178 (14)
W2—O11	1.866 (7)	N1—C5	1.493 (14)
W2—O9	1.951 (7)	N1—C2	1.524 (14)
W2—O6	1.961 (7)	N1—H1NB	0.9200
W2—O1	2.384 (7)	N1—H1NA	0.9200
W3—O31	1.713 (7)	N2—C7	1.540 (13)
W3—O16	1.892 (8)	N2—C10	1.541 (13)
W3—O12	1.894 (8)	N2—H2NB	0.9200
W3—O10	1.935 (8)	N2—H2NA	0.9200
W3—O5	1.937 (7)	N3—C15	1.485 (15)
W3—O2	2.385 (6)	N3—C12	1.502 (14)
W4—O32	1.713 (7)	N3—H3NB	0.9200
W4—O13	1.869 (7)	N3—H3NA	0.9200
W4—O17	1.898 (7)	N4—C20	1.504 (14)
W4—O7	1.926 (7)	N4—C17	1.516 (14)
W4—O11	1.961 (7)	N4—H4NB	0.9200
W4—O1	2.334 (6)	N4—H4NA	0.9200
W5—O33	1.713 (7)	N5—C22	1.460 (15)
W5—O8	1.886 (7)	N5—C25	1.522 (14)
W5—O14	1.897 (7)	N5—H5NB	0.9200
W5—O12	1.900 (8)	N5—H5NA	0.9200
W5—O18	1.935 (7)	C1—C2	1.516 (15)
W5—O3	2.369 (6)	C2—C3	1.534 (14)
W6—O34	1.711 (6)	C2—H2A	1.0000
W6—O9	1.854 (7)	C3—C4	1.537 (14)
W6—O10	1.898 (7)	C3—H3A	0.9900
W6—O19	1.921 (7)	C3—H3B	0.9900
W6—O20	1.942 (8)	C4—C5	1.523 (16)
W6—O2	2.325 (7)	C4—H4A	0.9900
W7—O35	1.711 (7)	C4—H4B	0.9900
W7—O23	1.883 (7)	C5—H5A	0.9900
W7—O24	1.892 (7)	C5—H5B	0.9900
W7—O13	1.913 (7)	C6—C7	1.511 (16)
W7—O14	1.920 (7)	C7—C8	1.559 (14)
W7—O3	2.400 (7)	C7—H7A	1.0000
W8—O36	1.697 (7)	C8—C9	1.533 (17)
W8—O19	1.868 (7)	C8—H8A	0.9900
W8—O25	1.906 (7)	C8—H8B	0.9900
W8—O15	1.916 (8)	C9—C10	1.505 (15)
W8—O21	1.939 (7)	C9—H9A	0.9900
W8—O4	2.359 (6)	C9—H9B	0.9900
W9—O37	1.715 (7)	C10—H10A	0.9900
W9—O26	1.887 (7)	C10—H10B	0.9900
W9—O20	1.903 (8)	C11—C12	1.487 (14)
W9—O22	1.904 (7)	C12—C13	1.542 (15)
W9—O16	1.937 (7)	C12—H12A	1.0000
W9—O2	2.373 (7)	C13—C14	1.552 (17)
W10—O38	1.721 (6)	C13—H13A	0.9900
W10—O17	1.887 (7)	C13—H13B	0.9900
W10—O23	1.889 (7)	C14—C15	1.516 (18)
W10—O27	1.920 (7)	C14—H14A	0.9900
W10—O21	1.934 (7)	C14—H14B	0.9900
W10—O4	2.354 (7)	C15—H15A	0.9900
W11—O39	1.730 (7)	C15—H15B	0.9900
W11—O18	1.901 (7)	C16—C17	1.494 (15)
W11—O22	1.911 (7)	C17—C18	1.492 (17)
W11—O24	1.919 (7)	C17—H17A	1.0000
W11—O28	1.921 (7)	C18—C19	1.49 (2)
W11—O3	2.341 (7)	C18—H18A	0.9900
W12—O40	1.725 (7)	C18—H18B	0.9900
W12—O28	1.866 (6)	C19—C20	1.47 (2)
W12—O27	1.888 (7)	C19—H19A	0.9900
W12—O26	1.900 (7)	C19—H19B	0.9900
W12—O25	1.936 (7)	C20—H20A	0.9900
W12—O4	2.385 (7)	C20—H20B	0.9900
B1—O3	1.503 (12)	C21—C22	1.525 (17)
B1—O4	1.513 (12)	C22—C23	1.52 (2)
B1—O1	1.534 (13)	C22—H22A	1.0000
B1—O2	1.556 (12)	C23—C24	1.49 (2)
O1S—C1	1.291 (14)	C23—H23A	0.9900
O1S—H1O	0.7151	C23—H23B	0.9900
O2S—C1	1.206 (15)	C24—C25	1.529 (18)
O3S—C6	1.232 (14)	C24—H24A	0.9900
O4S—C6	1.310 (14)	C24—H24B	0.9900
O4S—H4O	0.9251	C25—H25A	0.9900
O5S—C11	1.243 (14)	C25—H25B	0.9900
			
O29—W1—O5	101.7 (3)	B1—O3—W7	125.0 (5)
O29—W1—O8	101.2 (3)	W11—O3—W7	88.5 (2)
O5—W1—O8	88.1 (3)	W5—O3—W7	88.2 (2)
O29—W1—O6	97.7 (3)	B1—O4—W10	124.4 (5)
O5—W1—O6	92.0 (3)	B1—O4—W8	126.9 (6)
O8—W1—O6	160.7 (3)	W10—O4—W8	89.9 (2)
O29—W1—O7	96.7 (3)	B1—O4—W12	126.4 (6)
O5—W1—O7	161.4 (3)	W10—O4—W12	88.7 (2)
O8—W1—O7	86.1 (3)	W8—O4—W12	88.5 (2)
O6—W1—O7	87.7 (3)	C6—O4S—H4O	107.0
O29—W1—O1	169.8 (3)	W1—O5—W3	148.7 (4)
O5—W1—O1	86.9 (3)	W1—O6—W2	118.9 (3)
O8—W1—O1	84.4 (3)	C11—O6S—H6O	119.8
O6—W1—O1	76.3 (3)	W4—O7—W1	118.7 (4)
O7—W1—O1	75.0 (3)	C16—O7S—H7O	109.5
O30—W2—O15	101.9 (3)	W5—O8—W1	147.7 (4)
O30—W2—O11	101.3 (3)	W6—O9—W2	149.7 (4)
O15—W2—O11	95.0 (3)	C21—O9S—H9O	103.3
O30—W2—O9	99.5 (3)	W6—O10—W3	118.9 (4)
O15—W2—O9	84.9 (3)	W2—O11—W4	119.5 (3)
O11—W2—O9	158.8 (3)	W3—O12—W5	148.9 (4)
O30—W2—O6	96.4 (3)	W4—O13—W7	148.6 (4)
O15—W2—O6	160.4 (3)	W5—O14—W7	120.8 (3)
O11—W2—O6	88.2 (3)	W2—O15—W8	149.8 (4)
O9—W2—O6	85.2 (3)	W3—O16—W9	120.5 (4)
O30—W2—O1	170.9 (3)	W10—O17—W4	147.1 (4)
O15—W2—O1	87.0 (3)	W11—O18—W5	119.7 (4)
O11—W2—O1	76.0 (3)	W8—O19—W6	147.5 (4)
O9—W2—O1	82.9 (3)	W9—O20—W6	118.7 (4)
O6—W2—O1	75.0 (2)	W10—O21—W8	118.6 (3)
O31—W3—O16	101.8 (4)	W9—O22—W11	148.5 (4)
O31—W3—O12	100.7 (4)	W7—O23—W10	150.2 (5)
O16—W3—O12	90.4 (3)	W7—O24—W11	120.4 (4)
O31—W3—O10	99.3 (3)	W8—O25—W12	119.0 (4)
O16—W3—O10	89.1 (3)	W9—O26—W12	149.6 (4)
O12—W3—O10	159.7 (3)	W12—O27—W10	121.0 (4)
O31—W3—O5	99.1 (4)	W12—O28—W11	148.6 (4)
O16—W3—O5	159.1 (3)	C5—N1—C2	109.2 (8)
O12—W3—O5	87.1 (3)	C5—N1—H1NB	109.8
O10—W3—O5	86.2 (3)	C2—N1—H1NB	109.8
O31—W3—O2	173.6 (3)	C5—N1—H1NA	109.8
O16—W3—O2	75.6 (3)	C2—N1—H1NA	109.8
O12—W3—O2	85.3 (3)	H1NB—N1—H1NA	108.3
O10—W3—O2	74.9 (3)	C7—N2—C10	107.2 (8)
O5—W3—O2	83.5 (3)	C7—N2—H2NB	110.3
O32—W4—O13	100.8 (3)	C10—N2—H2NB	110.3
O32—W4—O17	100.8 (3)	C7—N2—H2NA	110.3
O13—W4—O17	87.7 (3)	C10—N2—H2NA	110.3
O32—W4—O7	97.6 (3)	H2NB—N2—H2NA	108.5
O13—W4—O7	92.3 (3)	C15—N3—C12	109.1 (9)
O17—W4—O7	161.2 (3)	C15—N3—H3NB	109.9
O32—W4—O11	96.7 (3)	C12—N3—H3NB	109.9
O13—W4—O11	162.4 (3)	C15—N3—H3NA	109.9
O17—W4—O11	87.2 (3)	C12—N3—H3NA	109.9
O7—W4—O11	87.1 (3)	H3NB—N3—H3NA	108.3
O32—W4—O1	170.0 (3)	C20—N4—C17	106.4 (9)
O13—W4—O1	87.3 (3)	C20—N4—H4NB	110.5
O17—W4—O1	85.3 (3)	C17—N4—H4NB	110.5
O7—W4—O1	75.9 (3)	C20—N4—H4NA	110.5
O11—W4—O1	75.5 (3)	C17—N4—H4NA	110.5
O33—W5—O8	101.7 (3)	H4NB—N4—H4NA	108.6
O33—W5—O14	97.0 (3)	C22—N5—C25	106.0 (9)
O8—W5—O14	91.7 (3)	C22—N5—H5NB	110.5
O33—W5—O12	102.2 (3)	C25—N5—H5NB	110.5
O8—W5—O12	87.8 (3)	C22—N5—H5NA	110.5
O14—W5—O12	160.5 (3)	C25—N5—H5NA	110.5
O33—W5—O18	97.6 (3)	H5NB—N5—H5NA	108.7
O8—W5—O18	160.6 (3)	O2S—C1—O1S	128.5 (11)
O14—W5—O18	88.0 (3)	O2S—C1—C2	123.2 (11)
O12—W5—O18	86.1 (3)	O1S—C1—C2	108.2 (10)
O33—W5—O3	169.6 (3)	C1—C2—N1	109.7 (9)
O8—W5—O3	86.4 (3)	C1—C2—C3	115.1 (9)
O14—W5—O3	76.1 (3)	N1—C2—C3	103.9 (8)
O12—W5—O3	84.4 (3)	C1—C2—H2A	109.3
O18—W5—O3	74.7 (3)	N1—C2—H2A	109.3
O34—W6—O9	101.3 (3)	C3—C2—H2A	109.3
O34—W6—O10	98.3 (3)	C2—C3—C4	104.2 (8)
O9—W6—O10	93.1 (3)	C2—C3—H3A	110.9
O34—W6—O19	99.3 (3)	C4—C3—H3A	110.9
O9—W6—O19	87.2 (3)	C2—C3—H3B	110.9
O10—W6—O19	161.9 (3)	C4—C3—H3B	110.9
O34—W6—O20	96.5 (4)	H3A—C3—H3B	108.9
O9—W6—O20	161.9 (3)	C5—C4—C3	102.8 (9)
O10—W6—O20	88.0 (3)	C5—C4—H4A	111.2
O19—W6—O20	86.2 (3)	C3—C4—H4A	111.2
O34—W6—O2	171.3 (3)	C5—C4—H4B	111.2
O9—W6—O2	86.5 (3)	C3—C4—H4B	111.2
O10—W6—O2	77.1 (3)	H4A—C4—H4B	109.1
O19—W6—O2	84.9 (3)	N1—C5—C4	104.8 (9)
O20—W6—O2	76.1 (3)	N1—C5—H5A	110.8
O35—W7—O23	102.9 (3)	C4—C5—H5A	110.8
O35—W7—O24	98.4 (3)	N1—C5—H5B	110.8
O23—W7—O24	91.8 (3)	C4—C5—H5B	110.8
O35—W7—O13	101.9 (3)	H5A—C5—H5B	108.9
O23—W7—O13	85.6 (3)	O3S—C6—O4S	125.6 (11)
O24—W7—O13	159.6 (3)	O3S—C6—C7	122.6 (10)
O35—W7—O14	98.0 (3)	O4S—C6—C7	111.7 (9)
O23—W7—O14	159.0 (3)	C6—C7—N2	107.9 (8)
O24—W7—O14	87.7 (3)	C6—C7—C8	112.2 (9)
O13—W7—O14	87.6 (3)	N2—C7—C8	103.2 (9)
O35—W7—O3	170.3 (3)	C6—C7—H7A	111.1
O23—W7—O3	84.7 (3)	N2—C7—H7A	111.1
O24—W7—O3	75.0 (3)	C8—C7—H7A	111.1
O13—W7—O3	84.6 (3)	C9—C8—C7	105.1 (9)
O14—W7—O3	74.9 (2)	C9—C8—H8A	110.7
O36—W8—O19	101.0 (3)	C7—C8—H8A	110.7
O36—W8—O25	96.1 (3)	C9—C8—H8B	110.7
O19—W8—O25	92.7 (3)	C7—C8—H8B	110.7
O36—W8—O15	101.4 (3)	H8A—C8—H8B	108.8
O19—W8—O15	86.9 (3)	C10—C9—C8	101.4 (9)
O25—W8—O15	162.2 (3)	C10—C9—H9A	111.5
O36—W8—O21	97.8 (3)	C8—C9—H9A	111.5
O19—W8—O21	161.1 (3)	C10—C9—H9B	111.5
O25—W8—O21	87.4 (3)	C8—C9—H9B	111.5
O15—W8—O21	87.3 (3)	H9A—C9—H9B	109.3
O36—W8—O4	170.4 (3)	C9—C10—N2	104.1 (8)
O19—W8—O4	86.0 (3)	C9—C10—H10A	110.9
O25—W8—O4	76.9 (3)	N2—C10—H10A	110.9
O15—W8—O4	85.4 (3)	C9—C10—H10B	110.9
O21—W8—O4	75.6 (3)	N2—C10—H10B	110.9
O37—W9—O26	101.6 (3)	H10A—C10—H10B	109.0
O37—W9—O20	99.3 (3)	O5S—C11—O6S	125.7 (10)
O26—W9—O20	92.5 (3)	O5S—C11—C12	122.1 (10)
O37—W9—O22	100.9 (3)	O6S—C11—C12	112.1 (9)
O26—W9—O22	86.7 (3)	C11—C12—N3	109.3 (9)
O20—W9—O22	159.5 (3)	C11—C12—C13	113.9 (9)
O37—W9—O16	97.7 (3)	N3—C12—C13	104.1 (9)
O26—W9—O16	160.2 (3)	C11—C12—H12A	109.8
O20—W9—O16	88.7 (3)	N3—C12—H12A	109.8
O22—W9—O16	85.3 (3)	C13—C12—H12A	109.8
O37—W9—O2	171.1 (3)	C12—C13—C14	104.4 (9)
O26—W9—O2	86.1 (3)	C12—C13—H13A	110.9
O20—W9—O2	75.6 (3)	C14—C13—H13A	110.9
O22—W9—O2	83.9 (3)	C12—C13—H13B	110.9
O16—W9—O2	75.1 (3)	C14—C13—H13B	110.9
O38—W10—O17	100.6 (3)	H13A—C13—H13B	108.9
O38—W10—O23	101.2 (3)	C15—C14—C13	101.0 (9)
O17—W10—O23	86.7 (3)	C15—C14—H14A	111.6
O38—W10—O27	98.1 (3)	C13—C14—H14A	111.6
O17—W10—O27	161.2 (3)	C15—C14—H14B	111.6
O23—W10—O27	88.2 (3)	C13—C14—H14B	111.6
O38—W10—O21	97.3 (3)	H14A—C14—H14B	109.4
O17—W10—O21	90.9 (3)	N3—C15—C14	103.6 (9)
O23—W10—O21	161.5 (3)	N3—C15—H15A	111.0
O27—W10—O21	88.2 (3)	C14—C15—H15A	111.0
O38—W10—O4	170.4 (3)	N3—C15—H15B	111.0
O17—W10—O4	86.3 (3)	C14—C15—H15B	111.0
O23—W10—O4	85.7 (3)	H15A—C15—H15B	109.0
O27—W10—O4	75.3 (3)	O8S—C16—O7S	125.4 (11)
O21—W10—O4	75.8 (3)	O8S—C16—C17	122.2 (11)
O39—W11—O18	99.4 (3)	O7S—C16—C17	112.3 (10)
O39—W11—O22	99.7 (3)	C18—C17—C16	117.0 (11)
O18—W11—O22	89.5 (3)	C18—C17—N4	104.0 (10)
O39—W11—O24	99.6 (3)	C16—C17—N4	110.1 (10)
O18—W11—O24	88.6 (3)	C18—C17—H17A	108.5
O22—W11—O24	160.6 (3)	C16—C17—H17A	108.5
O39—W11—O28	99.8 (3)	N4—C17—H17A	108.5
O18—W11—O28	160.8 (3)	C17—C18—C19	102.7 (11)
O22—W11—O28	87.2 (3)	C17—C18—H18A	111.2
O24—W11—O28	88.2 (3)	C19—C18—H18A	111.2
O39—W11—O3	173.6 (3)	C17—C18—H18B	111.2
O18—W11—O3	75.9 (3)	C19—C18—H18B	111.2
O22—W11—O3	84.8 (3)	H18A—C18—H18B	109.1
O24—W11—O3	76.0 (3)	C20—C19—C18	105.0 (13)
O28—W11—O3	84.9 (3)	C20—C19—H19A	110.8
O40—W12—O28	100.3 (4)	C18—C19—H19A	110.8
O40—W12—O27	99.0 (3)	C20—C19—H19B	110.8
O28—W12—O27	89.8 (3)	C18—C19—H19B	110.8
O40—W12—O26	101.7 (3)	H19A—C19—H19B	108.8
O28—W12—O26	87.1 (3)	C19—C20—N4	105.7 (11)
O27—W12—O26	159.3 (3)	C19—C20—H20A	110.6
O40—W12—O25	98.8 (3)	N4—C20—H20A	110.6
O28—W12—O25	160.9 (3)	C19—C20—H20B	110.6
O27—W12—O25	87.9 (3)	N4—C20—H20B	110.6
O26—W12—O25	88.4 (3)	H20A—C20—H20B	108.7
O40—W12—O4	171.8 (3)	O10S—C21—O9S	126.1 (11)
O28—W12—O4	85.5 (3)	O10S—C21—C22	123.8 (11)
O27—W12—O4	75.0 (3)	O9S—C21—C22	110.0 (10)
O26—W12—O4	84.3 (3)	N5—C22—C23	106.1 (11)
O25—W12—O4	75.7 (3)	N5—C22—C21	109.2 (10)
O3—B1—O4	110.3 (8)	C23—C22—C21	111.5 (13)
O3—B1—O1	111.0 (7)	N5—C22—H22A	110.0
O4—B1—O1	110.0 (7)	C23—C22—H22A	110.0
O3—B1—O2	108.8 (7)	C21—C22—H22A	110.0
O4—B1—O2	108.0 (7)	C24—C23—C22	106.9 (12)
O1—B1—O2	108.6 (7)	C24—C23—H23A	110.4
B1—O1—W4	124.8 (5)	C22—C23—H23A	110.4
B1—O1—W1	125.5 (5)	C24—C23—H23B	110.3
W4—O1—W1	90.4 (2)	C22—C23—H23B	110.3
B1—O1—W2	126.0 (5)	H23A—C23—H23B	108.6
W4—O1—W2	89.0 (2)	C23—C24—C25	104.0 (11)
W1—O1—W2	89.8 (2)	C23—C24—H24A	111.0
C1—O1S—H1O	120.6	C25—C24—H24A	111.0
B1—O2—W6	126.5 (5)	C23—C24—H24B	110.9
B1—O2—W9	125.5 (6)	C25—C24—H24B	111.0
W6—O2—W9	89.5 (2)	H24A—C24—H24B	109.0
B1—O2—W3	125.8 (5)	N5—C25—C24	101.4 (10)
W6—O2—W3	89.0 (2)	N5—C25—H25A	111.5
W9—O2—W3	88.7 (2)	C24—C25—H25A	111.5
B1—O3—W11	127.6 (6)	N5—C25—H25B	111.5
B1—O3—W5	125.7 (5)	C24—C25—H25B	111.5
W11—O3—W5	89.5 (2)	H25A—C25—H25B	109.3
			
O3—B1—O1—W4	59.2 (8)	O20—W6—O10—W3	74.5 (5)
O4—B1—O1—W4	−63.2 (8)	O2—W6—O10—W3	−1.7 (4)
O2—B1—O1—W4	178.8 (4)	O31—W3—O10—W6	−175.4 (5)
O3—B1—O1—W1	−61.3 (9)	O16—W3—O10—W6	−73.6 (5)
O4—B1—O1—W1	176.3 (5)	O12—W3—O10—W6	15.1 (13)
O2—B1—O1—W1	58.4 (8)	O5—W3—O10—W6	86.0 (5)
O3—B1—O1—W2	177.8 (5)	O2—W3—O10—W6	1.7 (4)
O4—B1—O1—W2	55.4 (9)	O30—W2—O11—W4	171.1 (4)
O2—B1—O1—W2	−62.6 (8)	O15—W2—O11—W4	−85.7 (4)
O32—W4—O1—B1	174.8 (15)	O9—W2—O11—W4	3.1 (11)
O13—W4—O1—B1	−41.5 (6)	O6—W2—O11—W4	75.0 (4)
O17—W4—O1—B1	46.5 (6)	O1—W2—O11—W4	0.0 (3)
O7—W4—O1—B1	−134.6 (6)	O32—W4—O11—W2	−173.5 (4)
O11—W4—O1—B1	134.8 (6)	O13—W4—O11—W2	12.4 (12)
O32—W4—O1—W1	−49.8 (16)	O17—W4—O11—W2	85.9 (4)
O13—W4—O1—W1	94.0 (3)	O7—W4—O11—W2	−76.1 (4)
O17—W4—O1—W1	−178.1 (3)	O1—W4—O11—W2	0.0 (3)
O7—W4—O1—W1	0.9 (2)	O31—W3—O12—W5	−130.1 (8)
O11—W4—O1—W1	−89.8 (3)	O16—W3—O12—W5	127.8 (8)
O32—W4—O1—W2	40.0 (16)	O10—W3—O12—W5	39.3 (15)
O13—W4—O1—W2	−176.3 (3)	O5—W3—O12—W5	−31.4 (8)
O17—W4—O1—W2	−88.3 (3)	O2—W3—O12—W5	52.3 (8)
O7—W4—O1—W2	90.6 (3)	O33—W5—O12—W3	133.7 (8)
O11—W4—O1—W2	0.0 (2)	O8—W5—O12—W3	32.2 (8)
O29—W1—O1—B1	170.5 (16)	O14—W5—O12—W3	−56.8 (15)
O5—W1—O1—B1	−41.8 (6)	O18—W5—O12—W3	−129.4 (9)
O8—W1—O1—B1	46.6 (6)	O3—W5—O12—W3	−54.4 (8)
O6—W1—O1—B1	−134.6 (7)	O32—W4—O13—W7	−135.7 (8)
O7—W1—O1—B1	134.1 (7)	O17—W4—O13—W7	−35.1 (8)
O29—W1—O1—W4	35.6 (18)	O7—W4—O13—W7	126.1 (8)
O5—W1—O1—W4	−176.7 (3)	O11—W4—O13—W7	38.3 (15)
O8—W1—O1—W4	−88.3 (3)	O1—W4—O13—W7	50.3 (8)
O6—W1—O1—W4	90.5 (3)	O35—W7—O13—W4	136.8 (8)
O7—W1—O1—W4	−0.9 (2)	O23—W7—O13—W4	34.5 (8)
O29—W1—O1—W2	−53.4 (18)	O24—W7—O13—W4	−48.9 (13)
O5—W1—O1—W2	94.3 (3)	O14—W7—O13—W4	−125.6 (8)
O8—W1—O1—W2	−177.3 (3)	O3—W7—O13—W4	−50.5 (8)
O6—W1—O1—W2	1.5 (3)	O33—W5—O14—W7	170.9 (4)
O7—W1—O1—W2	−89.9 (3)	O8—W5—O14—W7	−87.0 (4)
O15—W2—O1—B1	−37.9 (6)	O12—W5—O14—W7	1.3 (12)
O11—W2—O1—B1	−133.8 (7)	O18—W5—O14—W7	73.6 (4)
O9—W2—O1—B1	47.3 (6)	O3—W5—O14—W7	−1.1 (4)
O6—W2—O1—B1	134.3 (7)	O35—W7—O14—W5	−172.1 (4)
O15—W2—O1—W4	95.9 (3)	O23—W7—O14—W5	15.0 (12)
O11—W2—O1—W4	0.0 (2)	O24—W7—O14—W5	−74.0 (4)
O9—W2—O1—W4	−178.9 (3)	O13—W7—O14—W5	86.2 (4)
O6—W2—O1—W4	−91.9 (3)	O3—W7—O14—W5	1.1 (4)
O15—W2—O1—W1	−173.6 (3)	O30—W2—O15—W8	−136.4 (8)
O11—W2—O1—W1	90.4 (3)	O11—W2—O15—W8	121.0 (8)
O9—W2—O1—W1	−88.4 (3)	O9—W2—O15—W8	−37.7 (8)
O6—W2—O1—W1	−1.5 (3)	O6—W2—O15—W8	22.3 (15)
O3—B1—O2—W6	178.6 (5)	O1—W2—O15—W8	45.4 (8)
O4—B1—O2—W6	−61.6 (9)	O36—W8—O15—W2	139.6 (8)
O1—B1—O2—W6	57.6 (8)	O19—W8—O15—W2	39.0 (8)
O3—B1—O2—W9	−60.4 (8)	O25—W8—O15—W2	−50.1 (15)
O4—B1—O2—W9	59.4 (9)	O21—W8—O15—W2	−123.0 (8)
O1—B1—O2—W9	178.6 (4)	O4—W8—O15—W2	−47.2 (8)
O3—B1—O2—W3	58.2 (9)	O31—W3—O16—W9	170.8 (4)
O4—B1—O2—W3	178.0 (5)	O12—W3—O16—W9	−88.3 (5)
O1—B1—O2—W3	−62.8 (8)	O10—W3—O16—W9	71.4 (4)
O9—W6—O2—B1	−40.3 (7)	O5—W3—O16—W9	−5.5 (11)
O10—W6—O2—B1	−134.4 (7)	O2—W3—O16—W9	−3.3 (4)
O19—W6—O2—B1	47.2 (7)	O37—W9—O16—W3	−171.4 (4)
O20—W6—O2—B1	134.5 (7)	O26—W9—O16—W3	21.5 (12)
O9—W6—O2—W9	−176.1 (3)	O20—W9—O16—W3	−72.2 (5)
O10—W6—O2—W9	89.9 (3)	O22—W9—O16—W3	88.2 (5)
O19—W6—O2—W9	−88.6 (3)	O2—W9—O16—W3	3.3 (4)
O20—W6—O2—W9	−1.2 (2)	O38—W10—O17—W4	−135.0 (8)
O9—W6—O2—W3	95.2 (3)	O23—W10—O17—W4	−34.2 (8)
O10—W6—O2—W3	1.2 (3)	O27—W10—O17—W4	40.5 (15)
O19—W6—O2—W3	−177.3 (3)	O21—W10—O17—W4	127.4 (8)
O20—W6—O2—W3	−89.9 (3)	O4—W10—O17—W4	51.7 (8)
O26—W9—O2—B1	−41.6 (6)	O32—W4—O17—W10	135.2 (8)
O20—W9—O2—B1	−135.2 (6)	O13—W4—O17—W10	34.6 (8)
O22—W9—O2—B1	45.5 (6)	O7—W4—O17—W10	−55.9 (14)
O16—W9—O2—B1	132.3 (6)	O11—W4—O17—W10	−128.5 (8)
O26—W9—O2—W6	94.8 (3)	O1—W4—O17—W10	−52.8 (8)
O20—W9—O2—W6	1.2 (2)	O39—W11—O18—W5	171.9 (4)
O22—W9—O2—W6	−178.0 (3)	O22—W11—O18—W5	−88.3 (4)
O16—W9—O2—W6	−91.3 (3)	O24—W11—O18—W5	72.4 (4)
O26—W9—O2—W3	−176.2 (3)	O28—W11—O18—W5	−8.0 (12)
O20—W9—O2—W3	90.2 (3)	O3—W11—O18—W5	−3.6 (4)
O22—W9—O2—W3	−89.0 (3)	O33—W5—O18—W11	−169.4 (4)
O16—W9—O2—W3	−2.3 (3)	O8—W5—O18—W11	16.9 (12)
O16—W3—O2—B1	−132.1 (7)	O14—W5—O18—W11	−72.5 (4)
O12—W3—O2—B1	−40.4 (7)	O12—W5—O18—W11	88.9 (5)
O10—W3—O2—B1	134.9 (7)	O3—W5—O18—W11	3.5 (4)
O5—W3—O2—B1	47.1 (7)	O36—W8—O19—W6	−135.2 (7)
O16—W3—O2—W6	91.8 (3)	O25—W8—O19—W6	128.0 (7)
O12—W3—O2—W6	−176.5 (3)	O15—W8—O19—W6	−34.3 (7)
O10—W3—O2—W6	−1.2 (3)	O21—W8—O19—W6	38.0 (14)
O5—W3—O2—W6	−89.0 (3)	O4—W8—O19—W6	51.4 (7)
O16—W3—O2—W9	2.3 (3)	O34—W6—O19—W8	134.3 (7)
O12—W3—O2—W9	93.9 (3)	O9—W6—O19—W8	33.4 (7)
O10—W3—O2—W9	−90.7 (3)	O10—W6—O19—W8	−58.1 (14)
O5—W3—O2—W9	−178.5 (3)	O20—W6—O19—W8	−129.7 (7)
O4—B1—O3—W11	−58.8 (9)	O2—W6—O19—W8	−53.3 (7)
O1—B1—O3—W11	179.0 (5)	O37—W9—O20—W6	170.9 (4)
O2—B1—O3—W11	59.6 (9)	O26—W9—O20—W6	−87.0 (4)
O4—B1—O3—W5	178.4 (5)	O22—W9—O20—W6	0.4 (11)
O1—B1—O3—W5	56.2 (9)	O16—W9—O20—W6	73.3 (4)
O2—B1—O3—W5	−63.3 (9)	O2—W9—O20—W6	−1.7 (3)
O4—B1—O3—W7	61.2 (9)	O34—W6—O20—W9	−173.6 (4)
O1—B1—O3—W7	−61.0 (9)	O9—W6—O20—W9	18.3 (12)
O2—B1—O3—W7	179.5 (5)	O10—W6—O20—W9	−75.5 (4)
O18—W11—O3—B1	−134.5 (7)	O19—W6—O20—W9	87.4 (4)
O22—W11—O3—B1	−43.6 (7)	O2—W6—O20—W9	1.7 (3)
O24—W11—O3—B1	133.5 (7)	O38—W10—O21—W8	170.6 (4)
O28—W11—O3—B1	44.1 (7)	O17—W10—O21—W8	−88.6 (4)
O18—W11—O3—W5	2.5 (3)	O23—W10—O21—W8	−6.3 (13)
O22—W11—O3—W5	93.4 (3)	O27—W10—O21—W8	72.7 (4)
O24—W11—O3—W5	−89.5 (3)	O4—W10—O21—W8	−2.6 (4)
O28—W11—O3—W5	−178.9 (3)	O36—W8—O21—W10	−170.4 (4)
O18—W11—O3—W7	90.8 (3)	O19—W8—O21—W10	16.3 (12)
O22—W11—O3—W7	−178.4 (3)	O25—W8—O21—W10	−74.5 (4)
O24—W11—O3—W7	−1.3 (2)	O15—W8—O21—W10	88.5 (4)
O28—W11—O3—W7	−90.7 (3)	O4—W8—O21—W10	2.6 (4)
O33—W5—O3—B1	178.2 (15)	O37—W9—O22—W11	132.3 (8)
O8—W5—O3—B1	−39.8 (7)	O26—W9—O22—W11	31.2 (8)
O14—W5—O3—B1	−132.5 (7)	O20—W9—O22—W11	−57.2 (14)
O12—W5—O3—B1	48.4 (7)	O16—W9—O22—W11	−130.7 (8)
O18—W5—O3—B1	135.8 (7)	O2—W9—O22—W11	−55.2 (8)
O33—W5—O3—W11	39.9 (17)	O39—W11—O22—W9	−130.6 (8)
O8—W5—O3—W11	−178.1 (3)	O18—W11—O22—W9	129.9 (8)
O14—W5—O3—W11	89.3 (3)	O24—W11—O22—W9	45.6 (15)
O12—W5—O3—W11	−89.9 (3)	O28—W11—O22—W9	−31.1 (8)
O18—W5—O3—W11	−2.5 (3)	O3—W11—O22—W9	54.0 (8)
O33—W5—O3—W7	−48.6 (17)	O35—W7—O23—W10	−136.8 (9)
O8—W5—O3—W7	93.4 (3)	O24—W7—O23—W10	124.2 (9)
O14—W5—O3—W7	0.8 (2)	O13—W7—O23—W10	−35.5 (9)
O12—W5—O3—W7	−178.4 (3)	O14—W7—O23—W10	36.0 (16)
O18—W5—O3—W7	−91.0 (3)	O3—W7—O23—W10	49.4 (9)
O23—W7—O3—B1	−42.0 (7)	O38—W10—O23—W7	136.3 (9)
O24—W7—O3—B1	−135.3 (7)	O17—W10—O23—W7	36.1 (9)
O13—W7—O3—B1	44.1 (7)	O27—W10—O23—W7	−125.8 (9)
O14—W7—O3—B1	133.0 (7)	O21—W10—O23—W7	−46.9 (17)
O23—W7—O3—W11	94.6 (3)	O4—W10—O23—W7	−50.5 (9)
O24—W7—O3—W11	1.3 (2)	O35—W7—O24—W11	170.8 (4)
O13—W7—O3—W11	−179.3 (3)	O23—W7—O24—W11	−85.8 (4)
O14—W7—O3—W11	−90.4 (3)	O13—W7—O24—W11	−3.5 (11)
O23—W7—O3—W5	−175.8 (3)	O14—W7—O24—W11	73.1 (4)
O24—W7—O3—W5	90.9 (3)	O3—W7—O24—W11	−1.8 (3)
O13—W7—O3—W5	−89.7 (3)	O39—W11—O24—W7	−173.3 (4)
O14—W7—O3—W5	−0.8 (2)	O18—W11—O24—W7	−74.0 (4)
O3—B1—O4—W10	−62.4 (9)	O22—W11—O24—W7	10.6 (11)
O1—B1—O4—W10	60.4 (9)	O28—W11—O24—W7	87.1 (4)
O2—B1—O4—W10	178.8 (5)	O3—W11—O24—W7	1.9 (3)
O3—B1—O4—W8	176.7 (5)	O36—W8—O25—W12	173.2 (4)
O1—B1—O4—W8	−60.5 (9)	O19—W8—O25—W12	−85.4 (4)
O2—B1—O4—W8	57.8 (9)	O15—W8—O25—W12	2.8 (13)
O3—B1—O4—W12	55.8 (9)	O21—W8—O25—W12	75.6 (4)
O1—B1—O4—W12	178.6 (5)	O4—W8—O25—W12	−0.2 (4)
O2—B1—O4—W12	−63.1 (9)	O40—W12—O25—W8	−173.7 (4)
O17—W10—O4—B1	−43.0 (7)	O28—W12—O25—W8	8.4 (13)
O23—W10—O4—B1	43.9 (7)	O27—W12—O25—W8	−74.9 (4)
O27—W10—O4—B1	133.2 (7)	O26—W12—O25—W8	84.7 (5)
O21—W10—O4—B1	−134.9 (7)	O4—W12—O25—W8	0.2 (4)
O17—W10—O4—W8	93.7 (3)	O37—W9—O26—W12	−134.3 (9)
O23—W10—O4—W8	−179.3 (3)	O20—W9—O26—W12	125.6 (9)
O27—W10—O4—W8	−90.0 (3)	O22—W9—O26—W12	−33.9 (9)
O21—W10—O4—W8	1.9 (3)	O16—W9—O26—W12	32.6 (16)
O17—W10—O4—W12	−177.8 (3)	O2—W9—O26—W12	50.2 (9)
O23—W10—O4—W12	−90.8 (3)	O40—W12—O26—W9	134.5 (9)
O27—W10—O4—W12	−1.5 (2)	O28—W12—O26—W9	34.6 (9)
O21—W10—O4—W12	90.4 (3)	O27—W12—O26—W9	−47.3 (14)
O19—W8—O4—B1	−42.4 (7)	O25—W12—O26—W9	−126.9 (9)
O25—W8—O4—B1	−136.2 (7)	O4—W12—O26—W9	−51.2 (9)
O15—W8—O4—B1	44.7 (7)	O40—W12—O27—W10	172.1 (4)
O21—W8—O4—B1	133.1 (7)	O28—W12—O27—W10	−87.6 (4)
O19—W8—O4—W10	−177.4 (3)	O26—W12—O27—W10	−6.2 (11)
O25—W8—O4—W10	88.8 (3)	O25—W12—O27—W10	73.5 (4)
O15—W8—O4—W10	−90.3 (3)	O4—W12—O27—W10	−2.2 (3)
O21—W8—O4—W10	−1.9 (3)	O38—W10—O27—W12	−170.7 (4)
O36—W8—O4—W12	−43.6 (19)	O17—W10—O27—W12	13.8 (12)
O19—W8—O4—W12	93.8 (3)	O23—W10—O27—W12	88.3 (4)
O25—W8—O4—W12	0.1 (3)	O21—W10—O27—W12	−73.5 (4)
O15—W8—O4—W12	−179.0 (3)	O4—W10—O27—W12	2.2 (3)
O21—W8—O4—W12	−90.6 (3)	O40—W12—O28—W11	−134.7 (9)
O28—W12—O4—B1	−40.7 (7)	O27—W12—O28—W11	126.2 (9)
O27—W12—O4—B1	−131.8 (7)	O26—W12—O28—W11	−33.3 (9)
O26—W12—O4—B1	46.8 (7)	O25—W12—O28—W11	43.2 (16)
O25—W12—O4—B1	136.6 (7)	O4—W12—O28—W11	51.2 (9)
O28—W12—O4—W10	92.6 (3)	O39—W11—O28—W12	132.2 (9)
O27—W12—O4—W10	1.5 (2)	O18—W11—O28—W12	−47.9 (15)
O26—W12—O4—W10	−179.9 (3)	O22—W11—O28—W12	32.8 (9)
O25—W12—O4—W10	−90.1 (3)	O24—W11—O28—W12	−128.3 (9)
O27—W12—O4—W8	91.5 (3)	O3—W11—O28—W12	−52.2 (9)
O26—W12—O4—W8	−89.9 (3)	O2S—C1—C2—N1	−0.8 (14)
O25—W12—O4—W8	−0.1 (3)	O1S—C1—C2—N1	179.5 (8)
O29—W1—O5—W3	−133.0 (8)	O2S—C1—C2—C3	115.8 (12)
O8—W1—O5—W3	−31.9 (8)	O1S—C1—C2—C3	−63.9 (12)
O6—W1—O5—W3	128.7 (8)	C5—N1—C2—C1	130.7 (9)
O7—W1—O5—W3	40.0 (15)	C5—N1—C2—C3	7.2 (10)
O1—W1—O5—W3	52.6 (8)	C1—C2—C3—C4	−148.4 (10)
O31—W3—O5—W1	131.8 (8)	N1—C2—C3—C4	−28.5 (11)
O16—W3—O5—W1	−51.9 (13)	C2—C3—C4—C5	39.2 (11)
O12—W3—O5—W1	31.4 (8)	C2—N1—C5—C4	17.2 (11)
O10—W3—O5—W1	−129.4 (8)	C3—C4—C5—N1	−34.5 (10)
O2—W3—O5—W1	−54.1 (8)	O3S—C6—C7—N2	−8.5 (14)
O29—W1—O6—W2	169.5 (4)	O4S—C6—C7—N2	167.8 (8)
O5—W1—O6—W2	−88.4 (4)	O3S—C6—C7—C8	104.5 (12)
O8—W1—O6—W2	1.6 (12)	O4S—C6—C7—C8	−79.2 (11)
O7—W1—O6—W2	73.0 (4)	C10—N2—C7—C6	119.1 (9)
O1—W1—O6—W2	−2.1 (4)	C10—N2—C7—C8	0.2 (11)
O30—W2—O6—W1	−175.0 (4)	C6—C7—C8—C9	−90.3 (10)
O15—W2—O6—W1	25.9 (12)	N2—C7—C8—C9	25.6 (11)
O11—W2—O6—W1	−73.9 (4)	C7—C8—C9—C10	−42.1 (10)
O9—W2—O6—W1	85.9 (4)	C8—C9—C10—N2	41.5 (10)
O1—W2—O6—W1	2.0 (4)	C7—N2—C10—C9	−26.3 (11)
O32—W4—O7—W1	171.0 (4)	O5S—C11—C12—N3	−6.5 (14)
O13—W4—O7—W1	−87.8 (4)	O6S—C11—C12—N3	173.3 (9)
O17—W4—O7—W1	2.0 (12)	O5S—C11—C12—C13	109.4 (12)
O11—W4—O7—W1	74.6 (4)	O6S—C11—C12—C13	−70.8 (12)
O1—W4—O7—W1	−1.2 (3)	C15—N3—C12—C11	126.8 (9)
O29—W1—O7—W4	−172.7 (4)	C15—N3—C12—C13	4.8 (10)
O5—W1—O7—W4	14.3 (12)	C11—C12—C13—C14	−97.9 (10)
O8—W1—O7—W4	86.5 (4)	N3—C12—C13—C14	21.0 (10)
O6—W1—O7—W4	−75.2 (4)	C12—C13—C14—C15	−38.3 (10)
O1—W1—O7—W4	1.2 (3)	C12—N3—C15—C14	−29.4 (11)
O33—W5—O8—W1	−134.4 (7)	C13—C14—C15—N3	41.0 (10)
O14—W5—O8—W1	128.0 (7)	O8S—C16—C17—C18	113.8 (15)
O12—W5—O8—W1	−32.4 (7)	O7S—C16—C17—C18	−65.8 (16)
O18—W5—O8—W1	39.2 (14)	O8S—C16—C17—N4	−4.7 (16)
O3—W5—O8—W1	52.1 (7)	O7S—C16—C17—N4	175.7 (10)
O29—W1—O8—W5	134.2 (7)	C20—N4—C17—C18	19.8 (13)
O5—W1—O8—W5	32.7 (7)	C20—N4—C17—C16	145.9 (10)
O6—W1—O8—W5	−58.0 (13)	C16—C17—C18—C19	−158.2 (15)
O7—W1—O8—W5	−129.7 (7)	N4—C17—C18—C19	−36.5 (17)
O1—W1—O8—W5	−54.4 (7)	C17—C18—C19—C20	40 (2)
O34—W6—O9—W2	−132.2 (8)	C18—C19—C20—N4	−27.9 (19)
O10—W6—O9—W2	128.7 (8)	C17—N4—C20—C19	5.0 (15)
O19—W6—O9—W2	−33.2 (8)	C25—N5—C22—C23	24.6 (15)
O20—W6—O9—W2	35.6 (15)	C25—N5—C22—C21	144.8 (10)
O2—W6—O9—W2	51.8 (8)	O10S—C21—C22—N5	−16.2 (18)
O30—W2—O9—W6	135.2 (8)	O9S—C21—C22—N5	165.5 (10)
O15—W2—O9—W6	34.0 (8)	O10S—C21—C22—C23	100.7 (16)
O11—W2—O9—W6	−56.7 (13)	O9S—C21—C22—C23	−77.7 (15)
O6—W2—O9—W6	−129.1 (8)	N5—C22—C23—C24	−1(2)
O1—W2—O9—W6	−53.7 (8)	C21—C22—C23—C24	−119.4 (14)
O34—W6—O10—W3	170.8 (5)	C22—C23—C24—C25	−23.2 (19)
O9—W6—O10—W3	−87.3 (5)	C22—N5—C25—C24	−38.4 (11)
O19—W6—O10—W3	3.2 (14)	C23—C24—C25—N5	37.1 (14)

### Hydrogen-bond geometry (Å, °)

**Table:**

*D*—H···*A*	*D*—H	H···*A*	*D*···*A*	*D*—H···*A*
N1—H1NB···O12^i^	0.92	2.47	3.228 (12)	140
N1—H1NB···O16^i^	0.92	2.51	3.059 (11)	119
N1—H1NB···O18^i^	0.92	2.49	3.132 (12)	128
N1—H1NB···O22^i^	0.92	2.28	3.016 (11)	136
O1S—H1O···O38^ii^	0.72	2.18	2.669 (11)	126
N1—H1NA···O2S	0.92	2.24	2.725 (12)	112
N1—H1NA···O36^iii^	0.92	2.01	2.855 (12)	152
N2—H2NB···O3S	0.92	2.18	2.687 (12)	114
N2—H2NB···O40^iv^	0.92	1.93	2.744 (11)	146
N2—H2NA···O6^v^	0.92	1.90	2.810 (11)	170
O4S—H4O···O3W	0.92	1.73	2.647 (12)	170
N3—H3NB···O3S^ii^	0.92	2.18	2.909 (13)	135
N3—H3NA···O5S	0.92	2.16	2.672 (11)	114
N3—H3NA···O10S	0.92	2.05	2.856 (12)	145
O6S—H6O···O1W	0.84	1.88	2.638 (13)	149
N4—H4NB···O7	0.92	1.96	2.821 (13)	155
O7S—H7O···O21^vi^	0.84	1.87	2.689 (11)	165
N4—H4NA···O8S	0.92	2.29	2.701 (13)	107
N5—H5NB···O10S	0.92	2.26	2.709 (12)	109
N5—H5NB···O25^i^	0.92	2.20	3.005 (11)	146
O9S—H9O···O2W	0.89	1.86	2.651 (12)	147
N5—H5NA···O31	0.92	2.25	2.830 (12)	120
N5—H5NA···O8S^vii^	0.92	2.00	2.855 (13)	154

Symmetry codes: (i) −*x*+3/2, −*y*+1, *z*+1/2; (ii) *x*−1/2, −*y*+3/2, −*z*+1; (iii) *x*, *y*, *z*+1; (iv) −*x*+2, *y*+1/2, −*z*+1/2; (v) −*x*+1, *y*+1/2, −*z*+1/2; (vi) *x*−1/2, −*y*+3/2, −*z*; (vii) −*x*+1, *y*−1/2, −*z*+1/2.
